# The Impact of Second Primary Malignancies on Head and Neck Cancer Survivors: A Nationwide Cohort Study

**DOI:** 10.1371/journal.pone.0062116

**Published:** 2013-04-16

**Authors:** Li-Jen Liao, Hsu-Wen Chou, Chi-Te Wang, Chen-Shuan Chung, Mei-Shu Lai

**Affiliations:** 1 Institute of Epidemiology and Preventive Medicine, College of Public Health, National Taiwan University, Taipei, Taiwan; 2 Department of Otolaryngology, Far Eastern Memorial Hospital, Taipei, Taiwan; 3 Department of Internal Medicine, Far Eastern Memorial Hospital, Taipei, Taiwan; 4 Center of Comparative Effectiveness Research, National Center of Excellence for Clinical Trial and Research, National Taiwan University Hospital, Taipei, Taiwan; Roswell Park Cancer Institute, United States of America

## Abstract

**Background:**

Head and neck cancer (HNC) is associated with a high rate of developing second primary malignancies(SPMs). But the impact on survival remains poorly understood before. Therefore, we want to estimate the impact of SPMs on HNC survivors.

**Methods and Findings:**

Between 1986 and 2008, a total of 9,996 SPMs were recorded for 93,891 patients with an initial diagnosis of HNC by the Taiwan Cancer Registry. Patients were followed with national death registry database to 2011.Using the Kaplan–Meier method, a time-dependent covariate was employed to compare the survival rates between patients with and without SPMs. A Cox proportional hazards model that treated age and sex as confounders was used to examine the hazard ratios of SPMs. The relative survival rates were calculated using age- and sex-specific life tables for the population. Parametric mixture cure fraction models were then employed to estimate the percentage of cancer survivors who would be cured. Use of the Kaplan–Meier method showed that the crude survival rates differed significantly for patients with and patients without SPMs (log-rank test <0.01). For the results of Cox proportional hazards regression analysis, SPMs had a significant influence on survival rates with univariate (HR 2.59,95% CI 2.53to 2.65) and multivariate analysis (HR 2.34, 2.28 to 2.40). Patients with SPMs of nasopharyngeal carcinoma (NPC) had the highest cure rate at 39%, where as esophageal and lung cancer had the worst prognosis, with a cure rate of 11%.

**Conclusions:**

A worse prognosis was found for second primary cancer such as esophageal or lung cancer. Patients and healthcare providers must strongly consider and have a high clinical suspicion of these SPMs.

## Introduction

Studies that focus on cancer patients' follow-up care are required to improve effective survivorship care plans. Instead of the previous definition of a cancer survivor being a person who has remained disease-free for 5 years, the current definition begins at the moment of diagnosis to provide hope to newly diagnosedpeople and to encourage changes in doctor-patient communication in the context of cancer.[1] The topic of cancer survivorshipis becoming increasingly important in current cancer management.

Head and neck cancer (HNC; including cancer of thenasopharynx, oral cavity, oropharynx, larynx, or hypopharynx) is associated with a high likelihood of developing second primary malignancies(SPMs); the standardized incidence ratio(SIR) is approximately 2.18(95% CI, 2.15 to 2.21),[2] for which the most common sites are the head and neck region, esophagus, and lungs.[3] The risk factors, treatment modality and prognosis of HNC are quite different. For example, nasopharyngeal and oropharyngeal carcinomas are related to Epstein–Barr virus (EBV) and human papilloma virus (HPV) infection, respectively, which are generally managed with chemoradiation.[4], [5] Other HNCs, e.g. oral cavity and hypopharynx, were related to carcinogen exposure, such as tobacco use, smoking, alcohol drinking and betel nut chewing, which can be managed with surgery or chemo-radiotherapy.[6] According to the theory of field cancerization[7], patients with a HNC are especially susceptible to SPMs. Population-based cancer databases have documented the significant impact of SPMs on HNC[8]–[11].It has been estimated that approximately one-third of HNSCC deaths are attributable to SPMs[2]; therefore, estimating their impact on survival ratesis essential.

In population-based cancer studies, using relative survival methods is becoming the standard.[12]However, to date, no study has investigated how SPMs impact the survival rates of HNC survivors by using relative survival methods. The objective of this study is to quantitatively estimate the impact of SPMs on the survival rate of HNC patients.

## Materials and Methods

### Ethics

This study was approved by the Far Eastern Memorial Hospital Research Ethics Committee.

### Data source

The incidence of SPMs was obtained for 93 891 patients with an initial diagnosis of HNC, which included primary cancer originating in the oral cavity (ICD-9:140 to 145,excluding 142), oropharynx (ICD-9: 146 and 149), hypopharynx (ICD-9: 148), nasopharynx (ICD-9: 147), and larynx (ICD-9: 161), and other head and neck cancers (ICD-9 142 and 160), as reported to the Taiwan Cancer Registry (TCR, http://crs.cph.ntu.edu.tw/) between January 1, 1986, and December 31, 2008. The TCR was founded in 1979 and is financially supported by the National Department of Health for estimating the incidence of cancer in Taiwan. The TCR is a population-based cancer registry that contained 22 million people in 2003. Hospitals with at least 50 beds were obliged to submit information on newly diagnosed cancer patients to the TCR, which reimbursed the hospitals according to the number of cases reported to reduce the likelihood of these statistics being underreported.

All registry records in the TCR database are anonymous. Every case is registered with a unique identification number that can be linked to the National Death Database. All cancer registry databases of the TCR have been systemically converted to the International Classification of Diseases, Ninth Revision codes. People who were not identified using this process were, therefore, considered to be alive for the purpose of this study (passive follow-up). The coding of multiple primaries followed a common set of rules proposed by the International Agency for Research on Cancer (IARC).[13] HNC patients were excluded from analysis if they met the following criteria: (1) their birth date was missing from their record; (2) their death date or SPM diagnosis date preceded the diagnosis date of the first primary cancer; and (3) were aged <20 or >90 years. Subsequently, 93 891 HNC cases (80 010 men and 13 881women) were included in the survival analysis. SPMs were classified as synchronous (within 6 months of diagnosis of primary tumor) and metachronous (more than 6 months after primary).

### Statistical analysis

The database was analyzed using the STATA statistical software package (version 12.0). Descriptive statistics of demographic data were presented as the mean ± standard deviation or frequency (percentage). To avoid misclassification, a time-dependent covariate that allocates follow-up visits for each patient in the non-second cancer group until the second cancer occurrence was used to compare the survival rate of patients with and without SPMs.

The overall survival curves for patients with and without SPMs were calculated using the Kaplan–Meier method and then tested using the log-rank method. Differences between the groups were examined using hazard ratios obtained by a Cox proportional hazards model, which treated age and sex as confounders.

The relative survival rate, which is the ratio of the observed survival of cancer patients to the survival expected in the age and sex-matched population, which was calculated using age- and sex-specific life tables of the population. Relative survival was used as the input data for the cure models. A parametric mixture cure fraction model was fitted to estimate the cure fraction(π). The relative survival functions were predicted for various SPM sites.

## Results

Overall, 93 891 HNC cases (80 010 men and 13 881women) from the TCR between 1986 and 2008were included in the survival analysis. Patient and tumor characteristics are shown in summary in Table 1. When comparing occurrences by subsites in patient and tumor characteristics, including time of follow-up visits, age, and sex, we observed significant differences between subsites for all parameters, which reflects the heterogeneity of HNC across subsites. The mean age at diagnosis of the first primary cancer was 53.3±13.0 years. Patient age at diagnosis of the first cancer was higher for laryngeal cancer (63.9±11.5) and lower for NPC (49.8±12.7). The mean follow-up time for HNC patients was 5.3 years.

**Table 1:** Patient Characteristics Grouped According to Head and Neck Cancer Sites (n = 93 891, 1986 to 2008).

Sites of first cancer	Sex	No.	Mean age (dx)	Mean F/U (y)*	No SPM	5-yRS	SD	Cure fraction (π)
**NPC**	**All**	**27** **834**	**49.8(12.7)**	**6.5(5.8)**	**1545**	**59.2%**	**0.3%**	**0.41(0.01)** ε
**(ICD-9 147)**	Female	7365	48.9(13.1)	7.4(6.2)	439	65.6%	0.6%	0.44(0.01)ε
	Male	20 469	50.1(12.6)	6.1(5.7)	1106	56.9%	0.4%	0.41(0.01)ε
**Oral cancer**	**All**	**43** **381**	**52.8(12.3)**	**4.9(4.8)**	**5196**	**55.6%**	**0.3%**	**0.50(0.003)** ε
**(ICD-9 140–141, 143–145)**	Female	4248	58.8(14.9)	5.9(5.6)	455	64.2%	0.8%	0.63(0.01)ε
	Male	39 133	52.2(11.8)	4.8(4.7)	4741	54.7%	0.3%	0.48(0.003)ε
**Oropharyngeal cancer**	**All**	**4813**	**53.8(11.7)**	**3.7(4.5)**	**707**	**38.8%**	**0.8%**	**0.37(0.01)** ε
**(ICD-9 146, 149)**	Female	485	54.7(14.2)	6.2(5.9)	56	65.3%	2.4%	0.63(0.03)ε
	Male	4328	53.7(11.4)	3.5(4.3)	651	35.8%	0.8%	0.34(0.01)ε
**Hypopharyngeal cancer**	**All**	**7104**	**57.7(11.8)**	**3.0(4.0)**	**1090**	**28.6%**	**0.6%**	**0.25(0.01)** ε
**(ICD-9 146, 149)**	Female	220	60.5(13.2)	3.6(5.0)	39	34.3%	3.4%	0.30(0.04)ε
	Male	6884	57.6(11.8)	3.0(4.0)	1051	28.4%	0.6%	0.25(0.01)ε
**Laryngeal cancer**	**All**	**8144**	**63.9(11.5)**	**6.2(5.8)**	**1169**	**65.5%**	**0.7%**	**0.56(0.01)** Φ
**(ICD-9 161)**	Female	434	62.9(13.3)	7.4(6.0)	45	75.0%	2.5%	0.57(0.17)Φ
	Male	7710	64.0(11.4)	6.2(5.7)	1124	65.0%	0.7%	0.55(0.01)Φ
**Other cancers**	**All**	**2615**	**53.2(16.1)**	**7.2(6.1)**	**289**	**72.9%**	**1.0%**	**0.66(0.02)** ε
**(ICD-9 142, 160)**	Female	1129	50.9(16.4)	8.4(6.1)	111	84.7%	1.3%	0.67(0.12)Φ
	Male	1486	54.9(15.7)	6.3(5.9)	178	63.8%	1.4%	0.58(0.02)ε
All Sites	Total	93 891	53.3(13.0)	5.3(5.3)	9996	55.1%	0.2%	0.45(0.003)

* Diagnosis of first primary cancer; Abbreviations: SPM  =  second primary malignancy; RS  =  relative survival.

ε Weibull distribution and identity link.

Φ Gamma distribution and identity link.

**Figure 1:**
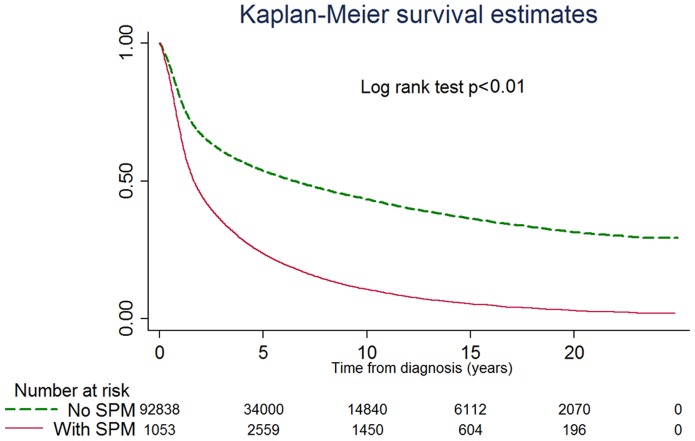
Actual survival rate estimated using the product-limit K-M method. The crude survival rate differed significantly for patients with or without SPM (log-rank test<0.01).

After managing SPMs as time-dependent covariates, the overall survival curves of patients with and without second cancers were calculated and plotted using the Kaplan–Meier method (Fig. 1), the crude survival rate differed significantly between patients with and without SPMs(log-rank test<0.01).

For the univariate Cox proportional hazards regression analysis results (Table 2), age (HR 1.02 95% CI 1.02 to 1.02), sex (HR 0.66, 0.64 to 0.68), and SPM (HR 2.59, 2.53 to 2.65) had a significant impact on patient survival. After adjusting for sex, the occurrence of SPMs still had a significant influence on the HN cancer survival rate (HR 2.34, 2.28 to 2.40).

**Table 2:** Univariate and Multivariate Cox Proportional Hazards Regression Analysis.

Univariate analysis					Multivariate analysis			
	Hazard ratio	95% CI		*p*-value	Hazard ratio	95% CI		*p*-value
**Sex(F/M)**	0.66	0.64-	0.68	<.001	0.71	0.69 -	0.73	<.001
**Age**	1.02	1.02 -	1.02	<.001	1.02	1.02 -	1.02	<.001
**Primary cancer**								
NPC	1.00				1.00			
Oral cancer	1.17	1.15 -	1.20	<.001	0.98	0.96 -	1.00	.021
Oropharyngeal cancer	1.78	1.72 -	1.85	<.001	1.42	1.37 -	1.48	<.001
Hypopharyngeal cancer	2.43	2.35 -	2.50	<.001	1.69	1.64 -	1.74	<.001
Laryngeal cancer	1.09	1.05 -	1.12	<.001	0.71	0.69 -	0.74	<.001
Other cancers	0.70	0.66 -	0.74	<.001	0.67	0.63 -	0.71	<.001
SPM*(yes/no)	2.59	2.53 -	2.65	<.001	2.34	2.28 -	2.40	<.001

Abbreviations: NPC  =  nasopharyngeal carcinoma; SPM  =  second primary malignancy

**Figure 2:**
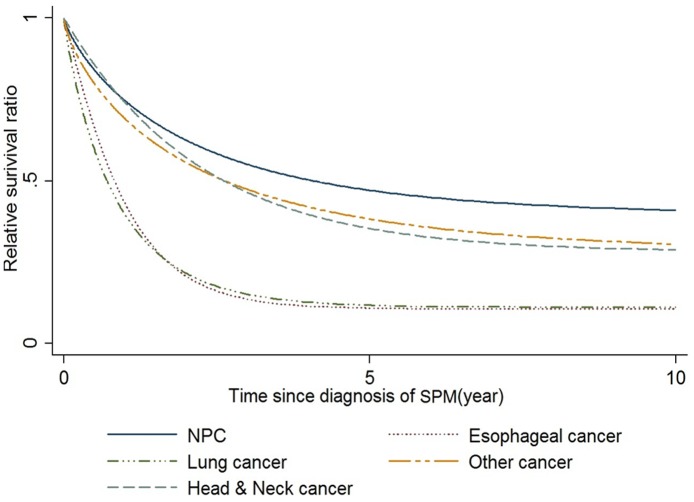
The estimated relative survival ratio with mixture cure modeling.

**Figure 3:**
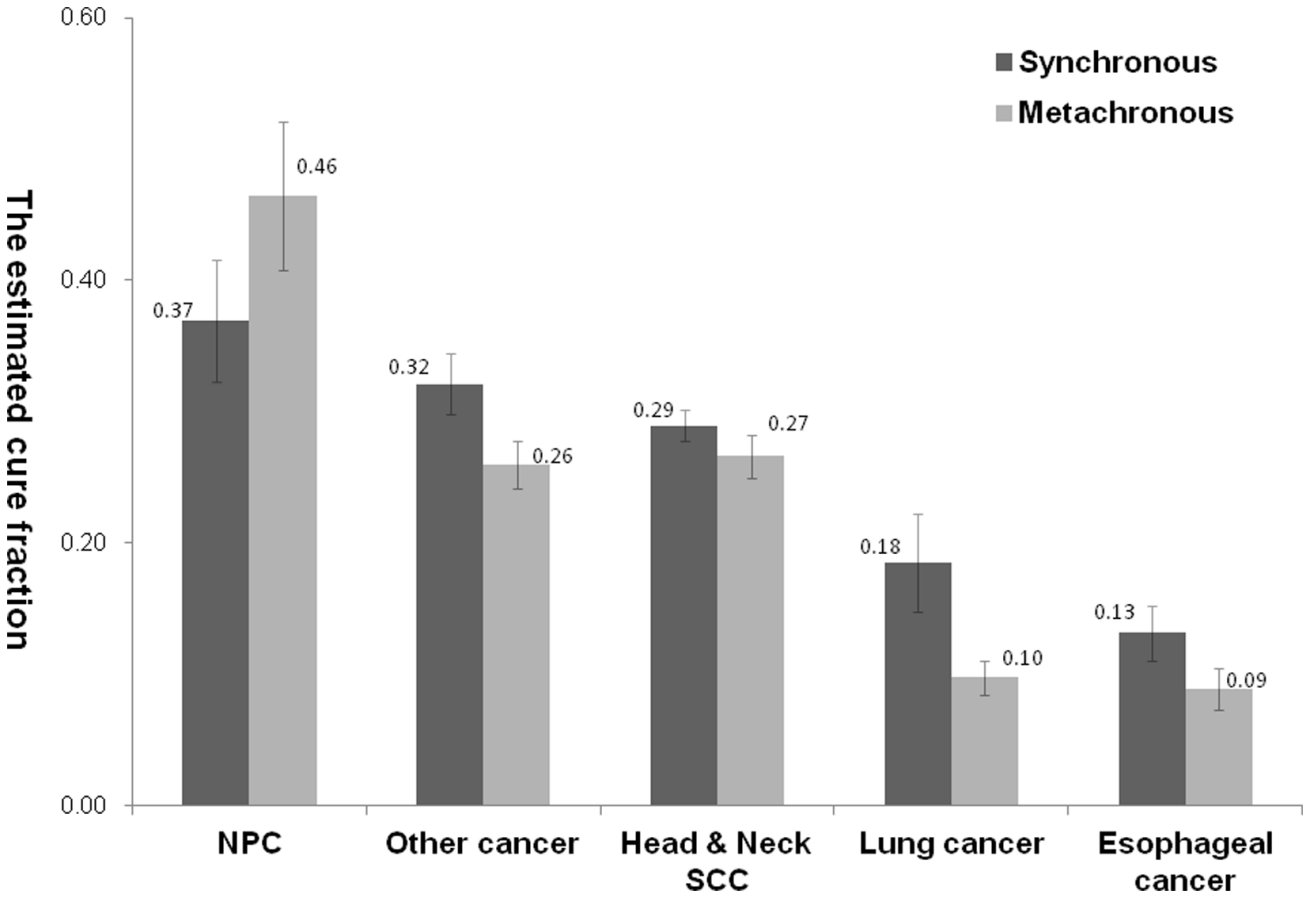
The estimated cure fraction with mixture cure modeling for synchronous versus metachronous SPM.

Overall,9996 of 93 891 HNC survivors developed SPMs. The mean time from the first cancer to the SPM was 2.71±3.95 years. The mean age of patients at diagnosis of SPM was 57.33±12.59 years. For the cure models, a mixture cure fraction model was fitted with parametric Weibullor gamma distribution and an identity link to estimate the cure fraction(π). The relative survival functions were predicted according to various SPM sites and are shown in Fig. 2. The cure fraction of various SPMs is shown in Table 3. Patients with an SPM of NPC had the best cure rate at 0.39±0.04, whereas the worst prognoses were of esophageal cancer and lung cancer, with a cure rate of 0.11±0.01.For SPMs such as NPC, the cure rate was better for metachronous(0.46±0.06) than synchronous diagnosis(0.37±0.05). For other SPMs, the cure rates were better for synchronous than metachronous diagnosis. (Figure 3)

**Table 3:** Results of mixture modeling the cure rate of various secondary primary malignancies.

SPM sites		Number	Duration	Age of SPM	Estimated cure fractionε(π)	
**NPC**	**311**		**1.03(2.21)**	**52.20(12.51)**	**0.39(0.04)**	
**H and N cancers**	**4902**		**1.78(3.21)**	**54.48(11.48)**	**0.28(0.01)**	
Oral vavity	3036		2.13(3.42)	53.32(11.31)	0.30(0.01)	
Oropharynx	720		1.27(2.79)	53.54(10.97)	0.25(0.02)	
Hypopharyn	619		1.20(2.71)	57.65(11.64)	0.24(0.02)	
Larynx	527		1.12(2.62)	58.72(11.32)	0.25(0.03)	
**Esophageal cancer**	**841**		**2.23(2.95)**	**56.02(10.75)**	**0.11(0.01)**	
**Lung cancer**	**856**		**4.66(4.55)**	**63.56(12.71)**	**0.11(0.01)**	
**Other cancers**	**3086**		**3.95(4.61)**	**61.00(13.11)**	**0.28(0.01)**	
Total	9996		2.71(3.95)	57.33(12.59)	0.26(0.01)	

Abbreviations: SPM  =  second primary malignancy.

ε Weibull distribution and identity link.

## Discussion

There are some factors might influence HNC survival. In presented study, we found sex, age, primary cancer sites and SPMs as significant prognostic factors of HNC survival. Among HNC, hypopharyngeal cancer is more difficult to catch in its earliest stages and has poor survival.[14] Another possible explanation could be the relatively weak association with alcohol and cigarette abuse and therefore differences in the prevalence of comorbidity in different gender and cancer subsites.[15] In the univariate analysis, the survival of oral cancer was poor than NPC with hazard ratio as 1.15–1.20; after adjusted age, sex and SPMs, the hazard ratio became non-significant (0.96–1.00). This finding indicated that age, sex and SPMs were potential confounding factors in survival analysis, therefore adjust these confounding factors with modeling is necessary.

HNC is associated with a high risk of SPMs, with an SIR of approximately 2.18(95% CI, 2.15 to 2.21),[2] for which the most common sites are the head and neck region, esophagus, and the lungs.[3] In this study, we measured the impact of SPMs on patient survival rates with a hazard ratio of 2.59 (95% CI 2.53 to 2.65) in univariate analysis and 2.34 (2.28 to 2.40) in multivariate analysis, and highlighted the importance of monitoring for SPMs in cancer patient care.

In population-based cancer studies, using relative survival methods is becoming the standard.[12] Relative survival is the ratio of the observed all-cause survival to the expected survival from a comparable group in the general population. If reliable information on the cause of death is available, cause-specific analysis can be conducted, where deaths not caused by the disease of interest are treated as censored observations. However, the cause of death may either not be recorded or obtained from death certificates, which are often recorded inaccurately.[16]However, the expected survival and/or the expected mortality rate can be obtained from national mortality statistics, and are typically calculated after matching for age, sex, year of diagnosis, and possibly other covariates.[17]

For most cancers, the relative survival curve appears to plateau after several years. This plateau effect occurs when the mortality rate of diseased patients is the same as the expected mortality rate of the average population, or the excess mortality rate is zero; that is, a population cure exists. Cancer studies may include a discussion on the proportion of patients cured of their disease, which is known as the cure fraction. One approach for modeling long-term survival studies is using mixture models, known as cure models in this study.[17], [18]

A cure model is a mixed model composed of the cure fraction model and the survival model of non-cured patients that estimates both the cure fraction and the survival function for non-cured patients. (Compared with traditional K-M method that considering only one variable at a time, cure model approach has the great advantage of simultaneously controlling multiple confounding factors, for example, age and gender in this study.) In population-based cancer studies, a cure is considered to have occurred when the mortality (hazard) rate for diseased patients equals that expected for the population. We used the STATA strsmix command[17], [19] for cure models that incorporate the background mortality rates in survival analysis.

Through this study, we found that a previous diagnosis of HNC has a significant impact on the incidence of secondary primary esophageal cancer.[20]A worse prognosis was found for second primary cancer as esophageal or lung cancer, with the cure rate as low as 0.1 in this study. In the national comprehensive cancer network (NCCN) guideline, [21] follow up of HNC patients with chest imaging is suggested in practice, but esophageal endoscopy has not been recommended before. Besides patients with SPMs of esophageal cancer (56.02±10.75y) tended to be younger than patients with SPMs of lung cancer (63.5±612.71y), HNC survivors should be also advised to screen for esophageal cancer. (Furthermore, our study had demonstrated a better survival on synchronous than metachronous SPMs, which may laso highlighted the benefit of early detection through adequate screening.) Conventional endoscopies and advanced image-enhanced endoscopies with biopsies are promising secondary prevention tools for identifying pre- and early malignant changes in esophageal mucosa.[22]Therefore, monitoring secondary primary esophageal pre- and early malignancy is essential for HNC survivor care.

Instead of the previous definition of a cancer survivor being a person who has remained cancer-free for 5 years, the current definition begins at the moment of diagnosis.[1] Thus, according to this definition, we incorporated all SPMs, including simultaneous, synchronous, and metachronous SPMs, into this study. In traditional definition, for SPMs such as NPC, the cure rate was better for metachronous(0.46±0.06) than synchronous diagnosis(0.37±0.05). For other SPMs, the cure rates were better for synchronous than metachronous diagnosis. (Figure 3) Our findings suggested the pathogenesis of NPC is different from other HNC because NPC was EBV related; while other HNC were resulted from environmental risk factors simultaneously exposure for the development of the primary malignancy and SPMs. HNC patients were supposed to live long enough to develop the SPMs as NPC. Therefore, for most HNC, the survival were better for synchronous than metachronous diagnosis due to earlier diagnosis and treatment of the second cancer. It also highlights the importance of surveillance of these SPMs for better outcome.

There are several limitations of the presenting study that needs to be addressed. First, although we have incorporated a nation-wide cancer registry to present the overall survival on the population, individual details were not routinely recorded, e.g. smoking, alcohol consumption, rural or urban locations and other associated socioeconomic status. Besides, the viral titers (e.g. EBV and HPV) and clinical-pathologic factors such as histology, grade, stage of SPMs were not recruited in this study, either. Further studies with more patient-level details are required to investigate the potential influence of these factors on the survival and management of SPMs on HNC.

## Conclusion

For head and neck cancer survivors, this study identified the presence of second primary cancer such as esophageal or lung cancer was associated with a worse prognosis. Patients and healthcare providers must strongly consider and have a high clinical suspicion of the potential influence of SPMs on survival.
